# Retraction Note: Posterior chamber phakic intraocular lens implantation after laser in situ keratomileusis

**DOI:** 10.1186/s40662-024-00400-6

**Published:** 2024-08-01

**Authors:** Kazutaka Kamiya, Kimiya Shimizu, Akihito Igarashi, Yoshihiro Kitazawa, Takashi Kojima, Tomoaki Nakamura, Kazuo Ichikawa, Sachiko Fukuoka, Kahoko Fujimoto, the Japan ICL Study Group

**Affiliations:** 1 Visual Physiology, School of Allied Health Sciences, Kitasato University, 1-15-1 Kitasato, Minami, Sagamihara, Kanagawa 252-0373 Japan; 2 Department of Ophthalmology, Sanno Hospital, Tokyo, Japan; 3 Sapia Tower Eye Clinic Tokyo, Tokyo, Japan; 4 Department of Ophthalmology, Keio University, Tokyo, Japan; 5 Nagoya Eye Clinic, Aichi, Japan; 6 Chukyo Eye Clinic, Aichi, Japan; 7 Department of Ophthalmology, Tane Memorial Eye Hospital, Osaka, Japan; 8 Fujimoto Eye Clinic, Osaka, Japan

**Retraction Note: Eye and Vision (2022) 9:15** 10.1186/s40662-022-00282-6

The authors have retracted this article because they did not obtain ethical approval for this study from every participating institution (Kitasato University Hospital, Sanno Hospital, Sapia Tower Eye Clinic Tokyo, Nagoya Eye Clinic, Chukyo Eye Clinic, Tane Memorial Eye Hospital, and Fujimoto Eye Clinic) in Japan. All authors agree to this retraction.

